# Contribution of DEAF1 Structural Domains to the Interaction with the Breast Cancer Oncogene LMO4

**DOI:** 10.1371/journal.pone.0039218

**Published:** 2012-06-19

**Authors:** Liza Cubeddu, Soumya Joseph, Derek J. Richard, Jacqueline M. Matthews

**Affiliations:** 1 School of Molecular Bioscience, The University of Sydney, Sydney, New South Wales, Australia; 2 Institute of Health and Biomedical Innovation, Queensland University of Technology, Kelvin Grove, Queensland, Australia; University of South Florida, United States of America

## Abstract

The proteins LMO4 and DEAF1 contribute to the proliferation of mammary epithelial cells. During breast cancer LMO4 is upregulated, affecting its interaction with other protein partners. This may set cells on a path to tumour formation. LMO4 and DEAF1 interact, but it is unknown how they cooperate to regulate cell proliferation. In this study, we identify a specific LMO4-binding domain in DEAF1. This domain contains an unstructured region that directly contacts LMO4, and a coiled coil that contains the DEAF1 nuclear export signal (NES). The coiled coil region can form tetramers and has the typical properties of a coiled coil domain. Using a simple cell-based assay, we show that LMO4 modulates the activity of the DEAF NES, causing nuclear accumulation of a construct containing the LMO4-interaction region of DEAF1.

## Introduction

The transcriptional co-regulator LMO4 (LIM-only protein 4) and the transcription factor DEAF1 (Deformed epidermal autoregulatory factor 1/NUDR/Suppressin) have several properties that suggest they act in common to regulate normal development and breast cancer. Both proteins are widely expressed but show high levels of expression in epithelial cells and the central nervous system (*e.g.*, [1], [2], [3], [4]), and mice knockouts of either gene result in similar brain, skeletal and cranial nerve defects [5]. In breast tissue, both LMO4 or DEAF1 are thought to play roles in cell proliferation and ductal side-branching [6], [7]. Their abilities to increase proliferation of mammary cells mark both proteins as potential contributors to breast tumour growth and metastasis.

LMO4 is present in all human breast tumour subtypes, with >50% of primary tumours showing increased levels of expression (*e.g.*, [8], [9], [10]), with a high level of nuclear LMO4 expression being associated with poor patient survival [9]. Forced overexpression of LMO4 causes mammary epithelial cells to proliferate *ex vivo*, increases mammary cell populations in a transgenic mouse model, and promotes cell invasion and tumour formation in human cell lines [9]. Although LMO4 contains little more than two protein-binding LIM domains, it can affect gene expression by modulating transcriptional events (*e.g.*, [11], [12], [13]), presumably by recruiting transcription factors, including DEAF1.

LMO4 and DEAF1 are co-expressed in breast tissue and were shown to interact in mammalian two-hybrid assays [4]. Given the potential functional significance of this interaction in breast cancer, we sought to understand how LMO4 and DEAF1 might cooperate to regulate cell proliferation. In this work, we used a combination of yeast two-hybrid, biophysical and cell-based assays to identify a tightly defined LMO4-binding region of DEAF1. This region contains (1) a specific LMO4-interaction domain (DEAF1_404–438_) and (2) the majority of a coiled coil domain (DEAF1_454–479_) encompassing the nuclear export signal (NES) of DEAF1. Further, we show that LMO4 can regulate the subcellular localisation of a DEAF1 construct incorporating the new LMO4-binding region. Together these results support the idea that high levels of LMO4 in the nucleus, which is a hallmark of sporadic breast cancers, may upset the delicate balance between interactions with partner proteins such as DEAF1.

## Results

### The LMO4 Minimal Binding Domain of DEAF1

**Figure 1:**
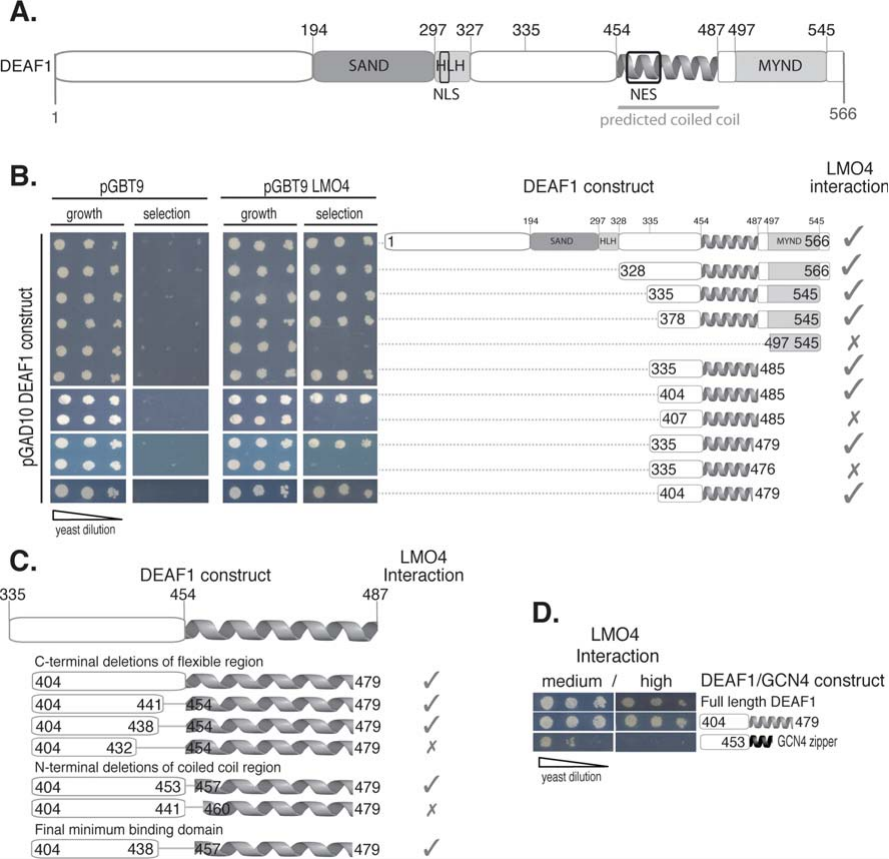
Establishing the LMO4-binding region of DEAF1. **A.** Schematic domain structure of the mouse DEAF1 protein containing a DNA-binding SAND (Sp100, AIRE-1, NucP41/75, DEAF11), a Helix-Loop-Helix (HLH) domain, a predicted coiled coil region (depicted as a helix), a protein-binding MYND (myeloid translocation protein 8, Nervy, DEAF1) domain, a nuclear localization signal (NLS) and a nuclear export signal (NES). The previously identified LMO4-binding region (335–545) [4] is indicated (thin grey line). **B.** Yeast two-hybrid experiments where *Saccharomyces cerevisiae* (AH109) were co-transformed with full-length DEAF1 fused to a transcriptional activator domain (pGAD10) and LMO4 fused to a DNA binding domain (pGBT9). Co-transformants were serially diluted and spotted on growth (−L/−W; growth) and high stringency interaction plates (−L/−W/−H/−A; selection). Left-most panels show controls. Schematic on right shows corresponding domain truncations of DEAF1 constructs used in the assays. Growth of yeast or its absence on selection plates indicates an interaction (ticks) or an abrogation of the interaction (crosses) with LMO4 respectively. **C.** DEAF1 internal deletion mutants were tested for interaction with LMO4 by yeast two-hybrid assays; interactions are represented as above. **D.** Yeast two-hybrid data for DEAF1/LMO4 interactions to assay replacement of the DEAF1 coiled coil domain by the dimeric GCN4 leucine zipper. Selection was medium/high stringency (−L/−W/−H+3AT)/(−L/−W/−H/−A). Yeast two-hybrid spot test results are shown. Three dilutions (A_600nm_ = 0.2, diluted serially 2×1-in-10) are spotted left to right to show differences in growth under each selection condition.

DEAF1 encodes a 566 residue (∼60 kDa) protein containing DNA-targetting SAND (Sp100/AIRE1/NucP41/75/DEAF1) domain and Helix-Loop-Helix (HLH) motifs at the N-terminus, a protein-binding MYND (Myeloid translocation protein 8/Nervy/DEAF1) domain at the C-terminus, a nuclear localisation sequence (NLS) and an NES (Figure 1A). Previous mammalian two-hybrid experiments showed that a large region encompassing the C-terminal half of DEAF1 (DEAF1_334–545_) could bind LMO4 [4]. That portion of DEAF1 contains various domains: a putative unstructured region, a putative coiled coil domain that overlaps with the NES [14], and the MYND domain. We aimed to define the LMO4-interaction domain by identifying the smallest region of DEAF1 that was sufficient to mediate an interaction by yeast two-hybrid analysis. Thus, we generated a series of DEAF1 truncation mutants, in which either the entire MYND domain was removed (to avoid generating mis-folded partial domains which can be “sticky” and give rise to non-specific interactions), and/or by systematically trimming the unstructured and coiled coil domains (where truncations are unlikely to have a major effect on structure; Figure 1B). DEAF1 truncations were fused to an N-terminal GAL4 activation domain where five residues separate GAL4 and the beginning of the DEAF1 constructs. Full length LMO4 was fused to an N-terminal GAL4 DNA binding domain. An interaction is inferred by yeast growth on selective media lacking essential nutrients (histidine or histidine and adenine) and/or the production of blue colour from α-X-gal (5-bromo-4-chloro-indolyl-α-D-galactopyranoside); the *His3*, *Ade2* and *Mel1* reporter genes are activated by a positive interaction. Although MYND domains are considered to be protein-binding motifs [15], the DEAF1-MYND domain was not involved in this interaction. Rather we identified a minimal LMO4-binding domain, DEAF1_404–479_, which includes 50 residues from the C-terminal end of the predicted unstructured region and the majority of the putative coiled coil.

Several partners of LMO and related LIM-homeodomain proteins interact with LIM domains through intrinsically unstructured domains of ∼30 residues [16], [17], and a coiled coil domain has previously been shown to stabilise an unstructured interaction domain in a yeast two-hybrid assay, but did not contribute to direct binding [18]. To test if the coiled coil domain (DEAF_454–479_) might stabilise a shorter region near the N-terminus of DEAF1_404–479_ for interaction with LMO4 we generated an additional set of internal deletions using the DEAF1_404–479_ template (Figure 1C). A 17-residue internal stretch of amino acids (DEAF1_439–456_) could be deleted without apparently affecting the interaction in the yeast two-hybrid assay. Fourteen of these residues were from the unstructured domain, and three from the putative coiled coil. Thus, within the contiguous DEAF1_404–479_ region, we identified DEAF1_404–438/457–479_ as the new binding motif for LMO4.

To test if the predicted coiled coil directly contacts LMO4, we compared the ability of LMO4 to bind the contiguous minimal LMO4-binding domain (DEAF1_404–479_), and a chimera, in which the DEAF1 coiled coil was replaced by the leucine zipper domain from GCN4, using yeast two-hybrid analysis (Figure 1D). Note that neither the isolated full length native DEAF1 coiled coil (DEAF1_454–487_) (data not shown), nor isolated the GCN4 leucine zipper [16] showed any detectable interaction with LMO4. The chimera in which the coiled coil domain from DEAF1 was replaced by the GCN4 leucine zipper showed clear evidence of an interaction with LMO4 under moderate, but not high stringency selection conditions, compared with the equivalent native construct, DEAF1_404–479_, which resulted in yeast growth under both moderate and high stringency conditions. The ability of the chimera to promote yeast growth under any condition suggested to us that the coiled coil was stabilising the rest of the construct rather than making specific interactions with LMO4, and that reduction in yeast growth was a result of less effective stabilisation by the GCN4 leucine zipper. However, it is also possible that the coiled coil domain from DEAF1 does make contacts with LMO4, which can be partially replaced by interactions with the leucine zipper domain of GCN4, or that self-association of DEAF1_404–438_ is required for binding. Although the leucine zipper domain of GCN4 is a highly characterised dimer (*e.g.,* [19]) the coiled coil domain from DEAF1 and its self-association properties have not been fully characterised.

### Self-association of DEAF1 by the Coiled Coil Domain

Full length DEAF1 was previously shown to self-associate using coimmunoprecipitation experiments. Wild type DEAF1 reinstates nuclear localisation of a DEAF1 mutant in which the nuclear localisation signal was disrupted, most likely through the *in vivo* association of wild type and mutant forms of DEAF1 [14]. Jensik and co-workers attributed this self-association, at least in part, to the putative coiled coil.

**Figure 2:**
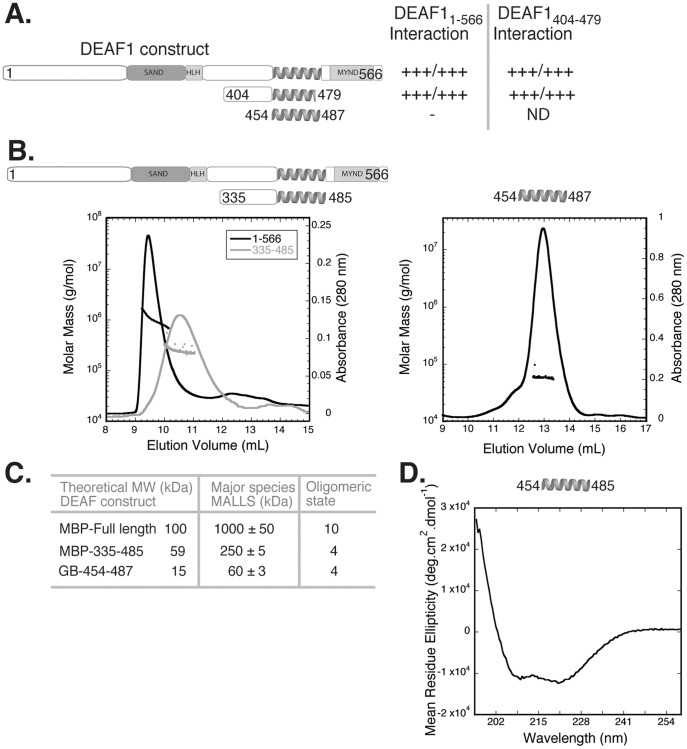
Characterising DEAF1 and the coiled coil domain. **A.** Schematic showing DEAF1 constructs used in yeast two-hybrid self-association experiments. Selection was medium/high stringency as in Figure 1D; +++ indicates strong growth, - indicates no growth, ND indicates not determined. **B.** SEC-MALLS analysis of full length and DEAF1_335–485_ constructs (left panel) and DEAF1_404–479_ and the coiled coil domain (right panel). DEAF1 proteins (∼200 µg) were applied to a Superose 12 column with an in line MALLS detector to determine weight-averaged molecular weight in solution. The elution (continuous line) and light-scattering (▪) are shown. **C.** Summary of the theoretical monomeric and experimentally determined molecular weight of DEAF1 proteins in A and B were used to calculate the oligomeric state. **D.** Far-UV circular dichroism spectropolarimetry (CD) spectrum of the DEAF1 coiled coil domain.

To characterise the self-association of DEAF1 we first used yeast two-hybrid analysis to establish that DEAF1_404–479_ (the new LMO4 binding region without internal deletions) could associate with both itself and full length DEAF1 (Figure 2A). The interaction with full length DEAF1 appeared weaker than that with DEAF1_404–479_ as judged by levels of growth under different stringency conditions. This difference in apparent binding strengths probably reflects differences in the stabilities of the DEAF1 constructs in yeast cells. Surprisingly, the isolated coiled coil construct (DEAF1_454–487_), was not able to interact with full length DEAF in this assay; however, only five residues separates the GAL4 activation domain and the beginning of the coiled coil in the construct. We postulate that the structured GAL4 activation domain may sterically prevent formation of the coiled coil.

To determine the oligomerisation state of DEAF1 and the coiled coil region *in vitro*, we made a series of DEAF1 constructs as recombinant proteins fused to the Maltose Binding Protein (MBP) or the β subunit of the Streptococcal G protein (GB_1_) tag. These proteins were subjected to size exclusion chromatography coupled to multi-angle laser light scattering (SEC-MALLS). This technique supplies information about weight average molecular weight in solution and requires relatively large quantities of essentially pure protein that cannot normally be obtained from mammalian cellular lysates. DEAF1 contains multiple protein domains, including the DNA-binding SAND and HLH domains that may also mediate self-association [20] and could complicate characterisation of the coiled coil domain. We tested a range of constructs, including full length DEAF1; DEAF1_335–485_, which lacks the SAND, HLH and MYND domains; the minimal LMO4-binding domain DEAF1_404–479_; and, the complete putative coiled-coil domain, DEAF1_454–487_. The larger constructs all showed evidence of self association, as indicated by weight average molecular weights corresponding to tetramers or higher order species (∼10 subunits for full length DEAF1, tetramer for DEAF1_335–485_), but sloping MALLS data indicating a mixture of species (Figure 2B) or small amounts of contaminating proteins precluded detailed analysis. In contrast, the isolated coiled coil, GB_1_-tagged DEAF1_454–487_ eluted from the size exclusion column as one major peak, with a molecular weight of ∼60 kDa, which corresponds to a tetramer (Figure 2C). Together these data indicate that DEAF1 can form tetramers through self-association of the coiled coil region, but other domains outside this region can also mediate self-association. We further subjected DEAF1_454–487_ to analysis by far-UV circular dichroism spectropolarimetry (CD) to probe the secondary structure of the isolated coiled coil (Figure 2D). The spectrum of the untagged protein is typical of α-helical conformation with minima at 208 and 222 nm, and a ratio of signal intensities at 208 nm:222 nm of <1 which is characteristic of coiled-coil domains (*e.g*., [21]). Together these data indicate that DEAF1_454–489_ forms a tetrameric coiled coil domain.

### LMO4 Blocks DEAF1 Nuclear Export Causing its Accumulation in the Nucleus

Eukaryotic NESs are often part of coiled coil domains due to their leucine-rich nature [22], [23], [24]. The location of the DEAF1 NES in the N-terminus of the coiled coil domain prompted the question: does binding by LMO4 affect the nuclear localisation of DEAF1? DEAF1 is a predominantly nuclear protein, and changes in its subcellular location to cytoplasmic, correlate with proliferative status of cells (*e.g.,* in colorectal carcinoma [25]). To begin to address this possibility, we developed a simple cell-based experiment that could show an effect on the nuclear localisation of transfected DEAF1_404–479_ in the absence and presence of transfected LMO4. We quantified nuclear translocation in HEK293 cells, which have low levels of endogenous LMO4 and DEAF1 proteins as determined by Western blotting (data not shown).

**Figure 3:**
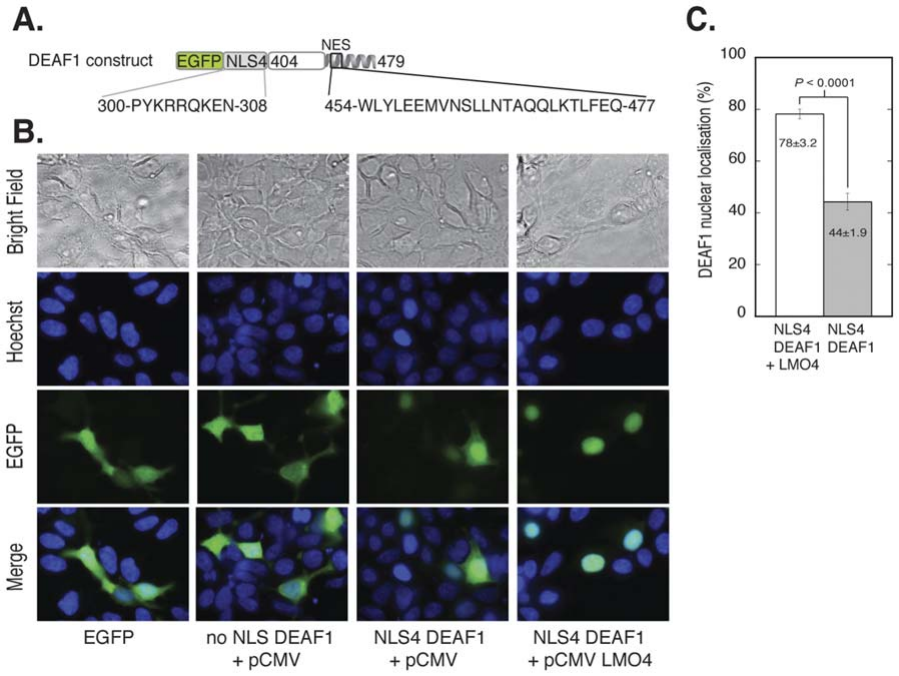
Nuclear localisation of EGFP-NLS4-DEAF1_404–479_ in the presence of LMO4. **A.** DEAF1 construct in pEGFP-C2 that was used for transfection. It has an N-terminal EGFP tag followed by the altered DEAF1 NLS4 and DEAF1_404–479._ The NLS4 and NES protein sequences and DEAF1 numbering are shown. **B.** HEK293 cells grown on cover slips in 6 well plates were transfected with a total of 4 µg of DNA: control pEGFP (panel 1), 2 µg EGFP-DEAF1_404–479_+2 µg empty pCMV vector (panel 2), 2 µg EGFP-NLS4-DEAF1_404–479_+2 µg empty pCMV (panel 3) and EGFP-NLS4-DEAF1_404–479_+2 µg pCMV LMO4 (panel 4). After 24 h transfection, cells were fixed with paraformaldehyde and nuclei stained with Hoechst dye. Cells were imaged for EGFP fluorescence (green) and nuclear staining (blue) by fluorescence microscopy. **C.** Quantification of A. The two-dimensional areas of *n* = 8 fields of view were measured for % nuclear localisation of EGFP-NLS4-DEAF1_404–479_ in the presence and absence of LMO4. Difference is statistically significant as *p*<0.05.

Using fluorescence microscopy of transiently transfected cells, we first demonstrated that DEAF1_404–479_ could be delivered to the nucleus using a well-characterised strong NLS from simian virus 40 large T-antigen (SV40) with an enhanced Green Fluorescent Protein (EGFP) tag. Transient transfection with EGFP-SV40-DEAF1_404–479_ showed essentially complete localisation of the construct to the nucleus, whereas EGFP-DEAF1_404–479_ (no NLS) was largely excluded from the nucleus (Figure S1A). Although these data demonstrated effective nuclear import of the DEAF1 construct, the presence of the strong SV40 NLS appeared to prevent net nuclear export via the DEAF1 NES. Thus, we engineered an NLS that allowed moderate nuclear localisation by replacing the SV40 NLS with sequences corresponding to the native DEAF1 NLS, or point mutants of these sequences. Utilising an extended version of the DEAF NLS [14] allowed for a larger choice of mutations than simply mutating the SV40 NLS. NLS1 contained the complete extended NLS. NLS2 contained the extended NLS with a glutamine in place of lysine at position 4, which has been shown to decrease the strength of an NLS [26]. NLS3 contained the short version of the DEAF1 NLS (nearly identical to SV40 NLS, thus strong). NLS4 was the short NLS with a glutamine substitution at position 4 (Figure S1B). Both NLS1 and NLS3 (the wildtype extended and short NLSs) efficiently directed DEAF1_404–479_ to the nucleus (87 and 85% nuclear accumulation, respectively), whereas NLS2 showed a reduced efficiency (48% nuclear) and NLS4 a markedly reduced efficiency (36%) compared to NLS1 and NLS3 (Figure S1C, D). GFP-NLS1-4 constructs without DEAF1_404–479_ were also tested for percent nuclear accumulation and showed the same trends (data not shown). We established that a construct comprising an enhanced Green Fluorescent Protein (EGFP) tag, the short NLS from DEAF1 with a glutamine in place of lysine at position 4 (NLS4), and DEAF1_404–479_ (EGFP-NLS4-DEAF1_404–479_), resulted in ∼40% nuclear localisation of the expressed protein (Figure S1). We used EGFP-NLS4-DEAF1_404–479_ (Figure 3A) to readily visualise and quantify changes in nuclear retention of transfected DEAF when co-transfected with LMO4.

The addition of pCMV-LMO4 to cells transfected with EGFP-NLS4-DEAF1_404–479_ had a marked effect on the nuclear localisation of EGFP-NLS4-DEAF1_404–479_; the GFP-tagged protein became predominantly localised in the nucleus in the presence of LMO4 (Figure 3B). The data shown were at plasmid ratios of 1∶1 DEAF:LMO4, (total of 4 µg plasmid) but the data were identical over all plasmid ratios tested (1∶1, 1∶1.5, 1∶2, data not shown), suggesting a relatively strong interaction between the two proteins. Quantification of DEAF1_404–479_ nuclear accumulation in Figure 3B, expressed as a percentage of cell area in two dimensions, showed a visually and statistically significant difference in the absence and presence of LMO4 (Figure 3C). Approximately 44% of GFP-NLS4-DEAF1_404–479_ is located in the nucleus in the absence of LMO4, but this nearly doubles to ∼78% when LMO4 is co-transfected. To control for the effect of LMO4 on nuclear localisation, we transfected EGFP-DEAF1_404–479_ (no NLS) with and without pCMV-LMO4 and showed that LMO4 had no effect on the nuclear localisation of DEAF1_404–479_ without an NLS; the EGFP protein appeared to be excluded from the nucleus (Figure S2). When we transfected EGFP-DEAF1_404–479_ (no NLS) in the presence of LMO4 we noted that DEAF1_404–479_ was concentrated to distinct foci around the periphery of the nucleus (Figure S2). This could indicate an LMO4-DEAF1_404–479_ interaction in the cytoplasm concentrated within these foci. Nuclear import with this construct should not be possible given the lack of an NLS, unless facilitated by additional binding partners within the cells.

## Discussion

Our data indicate that the DEAF1 coiled coil forms a tetramer *in vitro*, and contributes to a bipartite LMO4-binding motif (DEAF1_404–438/457–479_) in yeast two-hybrid assays. The native tetrameric coiled coil can be replaced by a non-native dimeric coiled coil with only a moderate loss of apparent affinity in this assay. Our current model for binding is that DEAF1_404–438_ makes direct contacts with LMO4 in a manner similar to other well characterised LMO and LIM-homeodomain binding domains [16], [17], [27], [28] and DEAF1_457–479_ either stabilises the construct, or provides an appropriate self-association state for the interaction with LMO4.

The presence of an NES in the coiled coil domain is not uncommon; NESs can be found in leucine rich segments of proteins, including coiled coil domains, located proximal to disordered regions [29], [30]. Leucine rich NESs from at least two different proteins bind the exportin protein CRM1 as helices. Conserved leucine (or other hydrophobic) residues that form the hydrophobic core of the coiled coil are critical for recognition by the exportin protein [31], [32]. The DEAF1 coiled coil sequence resembles a typical NES [24], suggesting that formation of a DEAF1 tetramer through the coiled coil domain would disfavour nuclear export. In this scenario the DEAF NES would only become available to exportins either by movement of the helix containing the NES, or monomerisation of individual helices. Although it is not yet clear if oligomerisation of DEAF1 is a requirement for the interaction with LMO4 *in vivo,* binding by LMO4 could prevent nuclear export of DEAF1 by stabilising DEAF1 tetramer (or higher order oligomers) through mass action effects, or by recruiting other protein partners that form oligomers and/or promote molecular clustering. Alternatively, LMO4 binding to DEAF1 may inhibit tetramerisation and prevent binding of an exportin to the DEAF NES, through direct steric inhibition, or by recruiting other partners that block binding. These hypotheses remain to be tested, although similar ‘masking effects’ of NESs have been shown for other proteins [33], [34], [35]. For example in the tetrameric form of p53 tumour suppressor, the NES lies within the tetramerisation domain and nuclear export appears to be regulated by the oligomerisation state of p53 [35]. Or in the case of the APC tumour suppressor where binding of CRM1 exportin and active–Ran allow movement of the helix containing the NES within the coiled coil moiety that then unmasks the NES [34]. An alternative explanation of our results is that DEAF1 could simply act as a nuclear localisation mechanism for LMO4. LMO4 is predominantly nuclear, although it does not have an NLS of its own. Other known binding partners of LMO4 all have NLS sequences, *e.g.,* LIM-domain binding protein 1 [36], CtBP-interacting protein ([37]) and oestrogen receptor α and metastasis-associated gene 1 [38]. LMO4 can therefore be transported into the nucleus by the most abundant/available partner, via a piggy-back mechanism. This is commonly seen for many NLS-deficient nuclear proteins (*e.g.,* [39], [40], [41]). Whichever is the case, the effect on either protein on the subcellular localisation of the other will modulate the activity of that protein, modulating the type and composition of complexes formed.

Our results begin to shed light on the mechanism of some breast cancers where high levels of LMO4 are present. Under these circumstances DEAF1 may become sequestered into nuclear LMO4-DEAF1 complexes, perturbing the normal functions of DEAF1. Forming aberrant transcription complexes can prevent the formation of normal complexes. For example Rac3 GTPase, which is linked to breast cancer, cellular migration and adhesion, is transcriptionally upregulated by DEAF1 in immortalised mammary epithelial cells [10]. Rac3 is a candidate target for LMO4:DEAF1 complexes. If these complexes are aberrantly forming due to the persistence of LMO4, it could provide a mechanism to increase cell proliferation and migration during breast oncogenesis. In contrast, the expression of hnRNP, a repression target of DEAF1 that is seen at high levels in some cancers [42], may be free to accumulate if other DEAF1 complexes are unable to form. Manipulating the interaction between LMO4 and DEAF1 to prevent the formation of aberrant transcriptional complexes may represent a potential novel therapeutic strategy with which to combat breast cancer.

## Materials and Methods

### Yeast Two-hybrid Assay

Mouse LMO4 (NCBI accession: NP_001155241) and DEAF1 (NM_016874) were used throughout. Full length DEAF1 was a gift from Jane Visvader. DEAF1 mutants and fusion constructs were generated by standard or optimised PCR methods [43]. Yeast two-hybrid assays were performed as described previously [16]. Co-transformants were serially diluted (A_600nm_ = 0.2, diluted serially 2×1-in-10) and spotted on selection plates for growth (−L/−W), medium (−L/−W/−H +1 mM 3-amino-1,2,4,-triazol (3-AT)) or high (−L/−W/−H/−A) stringency selection.

### Recombinant DEAF1 Protein Production

Full length DEAF1 and DEAF1_335–485_ were cloned into pMALC2 to produce an N-terminal maltose-binding protein (MBP) fusion when expressed in *Escherichia coli* Rosetta 2 strain (Novagen) induced with 0.1 mM isopropylthiogalactoside (IPTG), 20°C, 16 h. The MBP fusion was purified by amylose affinity chromatography in 20 mM Tris-HCl pH 8.0, 500 mM NaCl, 1 mM dithiothreitol (DTT) and eluted in 50 mM maltose. MBP fusion proteins were further purified by size exclusion chromatography (SEC) on a Superdex 200 column (GE Healthcare). The DEAF1_454–485_ construct was expressed in Rosetta 2 cells (induced with 0.4 mM IPTG, 30°C, 16 h) with an N-terminal His-GB1 (hexa-histidine/Gβ-1 domain of streptococcal protein G) tag [44]. This construct contains a 17-residue linker between the GB1 tag and DEAF1 to eliminate possible problems with steric hindrance. Protein was purified by batch anion exchange chromatography (DEAE sepharose, GE Healthcare) in 20 mM Tris-HCl pH 8.0, 100 mM NaCl, 1 mM DTT, 10% glycerol and eluted in 20 mM Tris-HCl pH 8.0, 600 mM NaCl, 20 mM imidazole, 5% glycerol, 1 mM 2-mercaptoethanol, 0.2 mM phenylmethylsulfonyl fluoride. Eluate was applied to a Ni-NTA resin (Invitrogen) equilibrated in the same buffer, eluted with 300 mM imidazole, and exchanged into 20 mM Tris-HCl pH 8.0, 150 mM NaCl and 1 mM DTT using a PD-10 desalting column (GE Healthcare). Sample purity was assessed by SDS-PAGE. The hexa-his-GB1 tag was cleaved from DEAF1_454–487_ using TEV protease (20°C, 16 h). Tag was removed by Ni-NTA affinity chromatography prior to SEC using a Superose 12 column (GE healthcare) to yield a 95% pure sample (as judged by SDS-PAGE).

### Protein Characterisation

Size exclusion chromatography coupled to multi-angle laser light scattering (SEC-MALLS) was carried out as described previously [17]. Far-UV circular dichroism spectropolarimetry (CD) was used to probe the secondary structure of DEAF1. DEAF1_454–487_ was dialysed into 20 mM Trisma pH 8.0, 50 mM NaF and 0.5 mM TCEP-HCl. Data were collected on a 20 µM sample using a Jasco J-720 spectropolarimeter at 20°C. The spectrum in Figure 2D represents the average of three accumulations collected at 20 nm/min and is buffer-baseline corrected.

### DEAF1 Nuclear Localisation

pEGFP-C2 and pCMV plasmids were from Clontech. The pEGFP-SV40 plasmid (enhanced Green Fluorescent Protein, EGFP, fused to the simian virus 40, SV40, large T-antigen NLS (PPKKKRKVEDP)) was a gift from Richard Grant. The SV40 NLS was replaced with synthetic DEAF1 NLS sequences to make pEGFP-NLS1–4. HEK293 cells (from ATCC) were cultured without antibiotics in high-glucose Dulbecco’s modified Eagle’s medium supplemented with 10% foetal bovine serum (Invitrogen) in a humidified 5% CO_2_ atmosphere at 37°C. For transfection experiments, HEK293 cells were grown on glass cover slips in 6-well plates. Transfection (4 µg of plasmid DNA) was carried out for 24 h using GeneJuice (Novagen) according to the manufacturer’s instructions. Adherent cells were washed with phosphate buffered saline pH 8 (PBS), fixed with 3% (wt/vol) paraformaldehyde, 2% sucrose in PBS, stained with Hoechst dye H33342 (which stains nuclei by binding AT-rich regions of DNA) and mounted onto class slides in ProLong Gold antifade medium (Invitrogen). Bright field and GFP fluorescent (FITC) images were obtained using a 100×objective on an Olympus BX51 System microscope and analySIS LS software (Olympus).

### Statistical Analysis of Cell Localisation

All data were derived from at least three independent experiments, with at least *n*  = 8 fields of view measured. Cell images were analysed using ImageJ image processing and analysis software, and the amount of EGFP protein in the cytoplasm and the nucleus was quantified as previously described [45]. Percent nuclear localisation ± s.e.m. was plotted in a bar graph in Fig 3B. Statistical significance was determined using Student’s *t*-test with *p*<0.05 considered significant.

## Supporting Information

Figure S1 **Choosing an appropriate NLS for DEAF1 nuclear localization experiments. A.** EGFP-DEAF1_404–479_ was first fused to the SV40 NLS. This strong NLS targeted all EGFP-DEAF1_404–479_ to the nucleus. **B.** Four EGFP-DEAF1_404–479_ constructs were made using variations to the native DEAF1 NLS to find a weaker NLS that did not target all DEAF1 to the nucleus. **C.** These EGFP-NLS-DEAF1_404–479_ constructs (4 µg) were transfected into HEK293 cells grown on cover slips in 6-well plates. After 24 h transfection, cells were fixed with paraformaldehyde and the nuclei stained with the Hoechst dye. Cells were imaged for EGFP fluorescence and nuclear staining by fluorescence microscopy. **D.** Quantification of percent nuclear accumulation of *n* = 6 fields of cells for each EGFP-DEAF-NLS construct used in C.
(TIF)

Figure S2 **Specificity of the LMO4 effect on EGFP-NLS4-DEAF1_404–479_ nuclear retention.** EGFP-DEAF1_404–479_ (no NLS, 2 µg) with and without LMO4 (2 µg) was transfected into HEK293 cells as stated previously. LMO4 has no effect on the nuclear localisation of DEAF1_404–479_ without an NLS. Although, in the presence of LMO4 this DEAF1_404–479_ (no NLS) construct appeared to concentrate to distinct foci around the periphery of the nucleus. This may indicate an LMO4-DEAF1_404–479_ interaction in the cytoplasm concentrated within these foci.
(TIF)
