# Supplementary figures and images for: Contribution of DEAF1 Structural Domains to the Interaction with the Breast Cancer Oncogene LMO4
Source: PLoS One. 2012 Jun 19;7(6):e39218. doi: 10.1371/journal.pone.0039218 (PMC3378519; doi:10.1371/journal.pone.0039218)

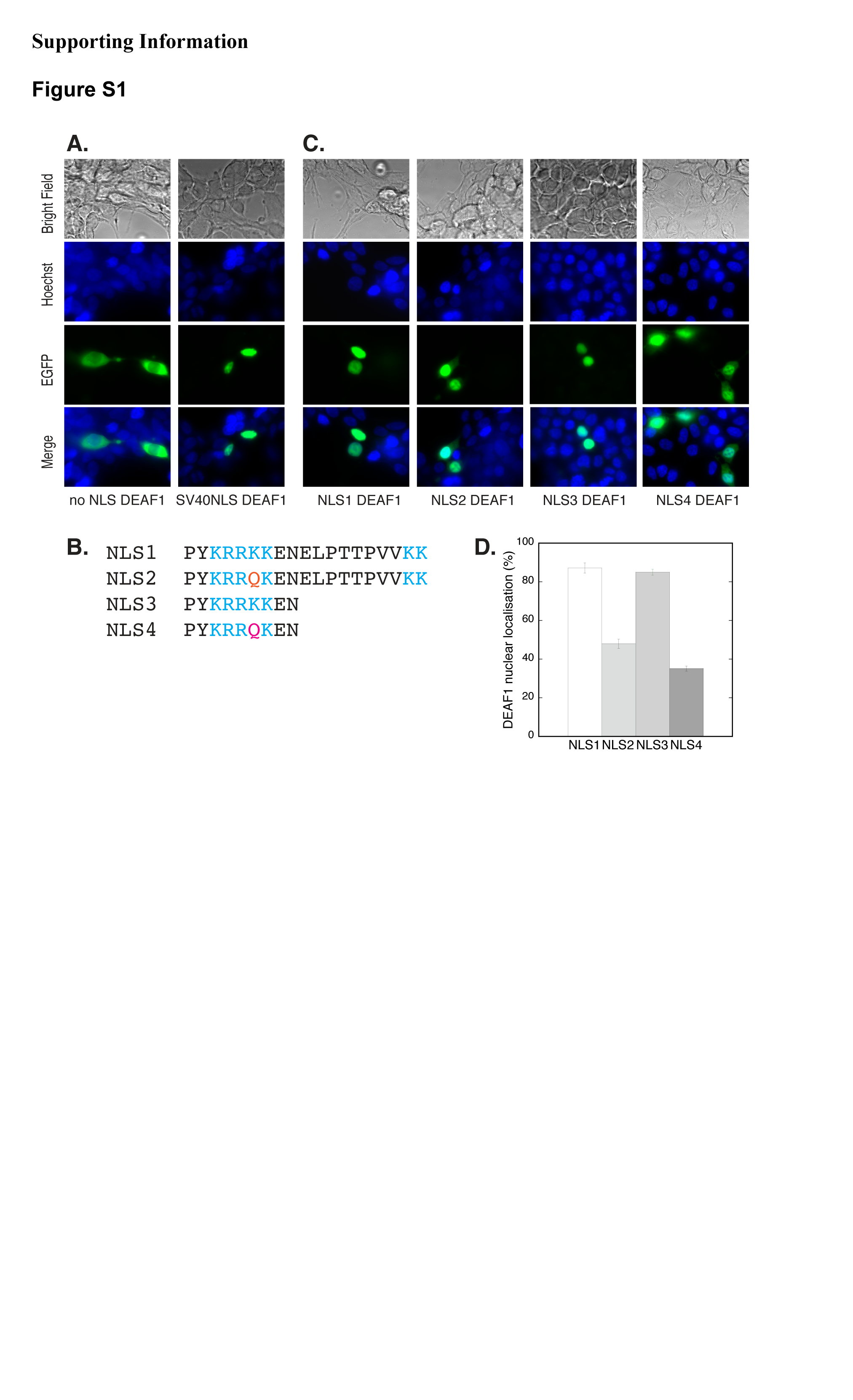

Supplement: Figure S1 — Choosing an appropriate NLS for DEAF1 nuclear localization experiments. A. EGFP-DEAF1404–479 was first fused to the SV40 NLS. This strong NLS targeted all EGFP-DEAF1404–479 to the nucleus. B. Four EGFP-DEAF1404–479 constructs were made using variations to the native DEAF1 NLS to find a weaker NLS that did not target all DEAF1 to the nucleus. C. These EGFP-NLS-DEAF1404–479 constructs (4 µg) were transfected into HEK293 cells grown on cover slips in 6-well plates. After 24 h transfection, cells were fixed with paraformaldehyde and the nuclei stained with the Hoechst dye. Cells were imaged for EGFP fluorescence and nuclear staining by fluorescence microscopy. D. Quantification of percent nuclear accumulation of n = 6 fields of cells for each EGFP-DEAF-NLS construct used in C. (TIF) [file pone.0039218.s001.tif]

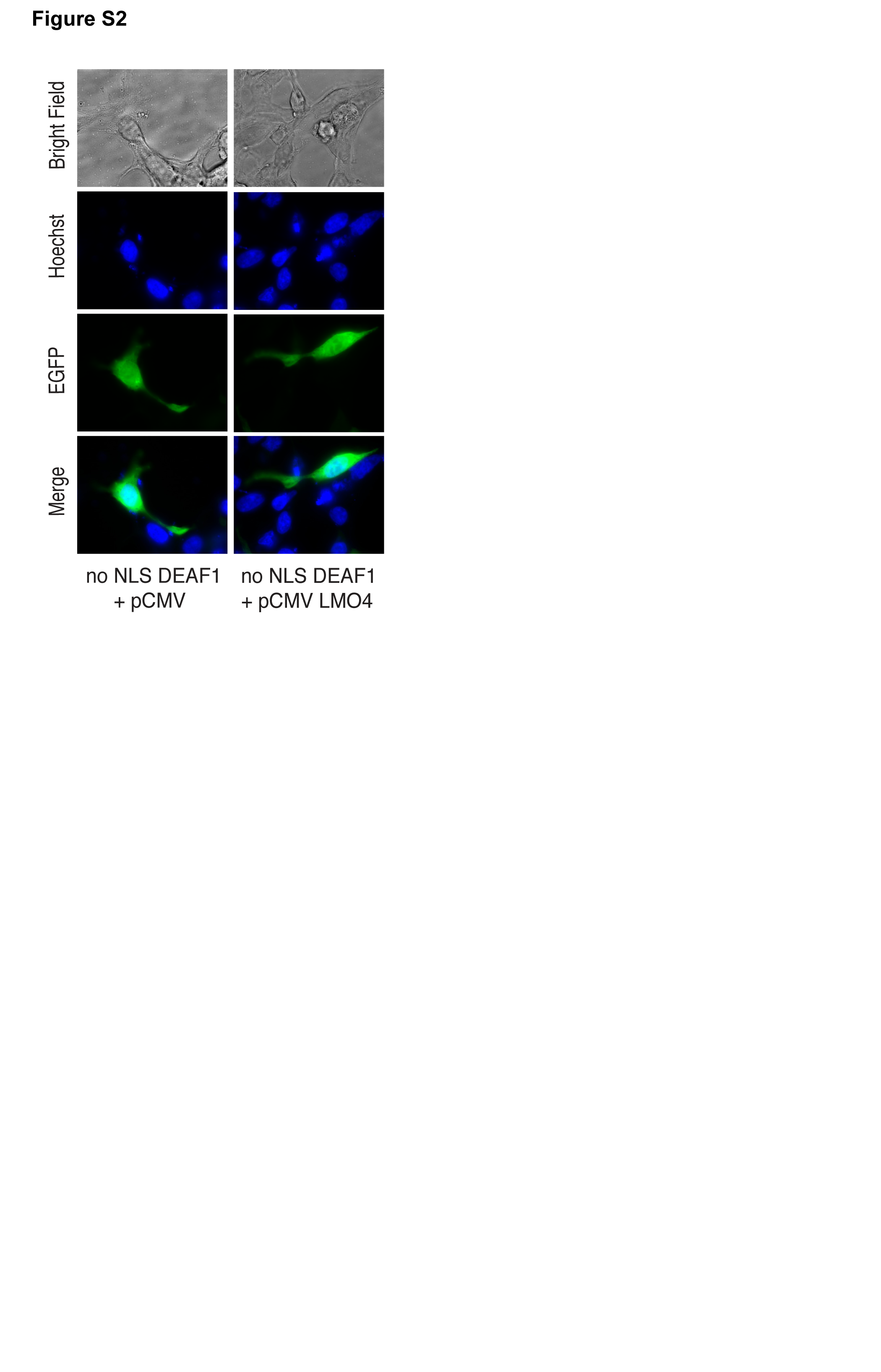

Supplement: Figure S2 — Specificity of the LMO4 effect on EGFP-NLS4-DEAF1404–479 nuclear retention. EGFP-DEAF1404–479 (no NLS, 2 µg) with and without LMO4 (2 µg) was transfected into HEK293 cells as stated previously. LMO4 has no effect on the nuclear localisation of DEAF1404–479 without an NLS. Although, in the presence of LMO4 this DEAF1404–479 (no NLS) construct appeared to concentrate to distinct foci around the periphery of the nucleus. This may indicate an LMO4-DEAF1404–479 interaction in the cytoplasm concentrated within these foci. (TIF) [file pone.0039218.s002.tif]
